# Anatomical Guideline for Retrobulbar Hyaluronidase Injection

**DOI:** 10.1111/jocd.70378

**Published:** 2025-08-05

**Authors:** Kyu‐Ho Yi, Jin‐Hyun Kim, Han Earl Lee, Jong Keun Song, Byungki Cho, Soo‐Bin Kim

**Affiliations:** ^1^ Division in Anatomy and Developmental Biology, Department of Oral Biology Human Identification Research Institute, BK21FOUR Project, Yonsei University College of Dentistry Seoul Korea; ^2^ You and I Clinic Seoul Korea; ^3^ Re & Re Plastic Surgery Clinic Seoul Korea; ^4^ Pixelab Plastic Surgery Clinic Seoul Korea; ^5^ It's Me Clinic Sejong Korea; ^6^ Institute of Biomaterial Implant, Department of Oral Anatomy, College of Dentistry Wonkwang University, Iksan 54538, Republic of Korea Iksan Republic of Korea

**Keywords:** cosmetic techniques, hyaluronic acid, ophthalmic artery, retrobulbar injections, vision disorders

## Abstract

**Background:**

Vision loss following cosmetic filler injections is a rare but devastating complication resulting from inadvertent intravascular embolization, most often affecting the ophthalmic artery. Retrobulbar hyaluronidase injection has been proposed as an emergency intervention, yet anatomical guidelines for its administration remain poorly defined.

**Objective:**

To propose an anatomically informed protocol for safe and effective retrobulbar hyaluronidase injection in cases of filler‐induced central retinal artery occlusion.

**Methods:**

Cadaveric dissections and micro‐CT imaging were used to map orbital anatomy, focusing on the spatial relationship between the optic nerve, ophthalmic artery, and adjacent structures. The inferolateral quadrant was identified as the safest trajectory for retrobulbar injection, minimizing risk to ocular muscles and nerves.

**Results:**

The optimal needle trajectory was from the inferolateral orbital rim toward the superior medial quadrant, avoiding critical neurovascular structures. A 35 mm, 22–23G needle was deemed appropriate for reaching the retrobulbar space while minimizing the risk of globe perforation.

**Conclusion:**

This study provides a standardized anatomical approach for retrobulbar hyaluronidase injection, aiming to improve safety and potentially restore perfusion in acute filler‐induced visual loss. Clinical implementation should proceed with caution and in multidisciplinary settings.

## 1 Introduction

The popularity of injectable cosmetic fillers has grown rapidly in recent years. Currently, dermal fillers rank as the second most commonly performed nonsurgical aesthetic procedure [1]. As the number of filler procedures increases, there has been a parallel rise in cases of retrograde embolization and associated ophthalmic ischemic events.

Filler‐related emboli have been shown to cause various types of orbital vascular occlusions, including ophthalmic artery occlusion (OAO), central retinal artery occlusion (CRAO), branch retinal artery occlusion (BRAO), among others [2, 3]. Studies have demonstrated that irreversible retinal injury—and consequent vision loss—can occur within 90 min of interrupted perfusion, highlighting the urgent need for timely intervention [4].

At present, there is no standardized first‐line treatment to promptly re‐establish ocular blood flow and prevent vision impairment resulting from filler‐induced ischemia. This article aims to outline a *relatively safer* anatomical technique for administering retrobulbar hyaluronidase in such scenarios.

## 2 Anatomical Factors

The leading theory behind ophthalmic complications following cosmetic filler injections involves retrograde embolization through arteries situated near the injection site—most commonly the supratrochlear, supraorbital, or dorsal nasal arteries. When filler is introduced into these peripheral arteries, the pressure of injection may exceed that of the arterial system, allowing the filler to move in a reverse direction toward the ophthalmic artery [5, 6].

Ischemic injury results not only from the direct blockage of blood flow by the embolus, but also from secondary processes such as local inflammation, platelet aggregation, and activation of the coagulation cascade. In certain cases, larger emboli or thrombi can break apart into smaller fragments, causing micro‐emboli that obstruct multiple distal branches of the ophthalmic artery, leading to multifocal ischemia [6, 7].

The specific manifestations of ischemic injury depend on which vascular territories are affected. When emboli become lodged in the central retinal or posterior ciliary arteries, the resulting perfusion failure can lead to retinal ischemia and vision loss, which may become irreversible if retinal cells undergo necrosis. Similarly, blockage of the posterior or anterior ciliary arteries can impair blood flow to the iris and ciliary body, resulting in anterior segment ischemia [8].

The type of filler used also influences the severity and pattern of ischemia. Visual complications have been documented with a range of injectables, including hyaluronic acid, platelet‐rich plasma (PRP), autologous fat, calcium hydroxyapatite, and poly‐(L)‐lactic acid (PLLA) used in periorbital rejuvenation [9, 10]. Among these, autologous fat tends to cause more proximal vascular blockages due to its larger particle size. In contrast, hyaluronic acid, with smaller particles, typically causes occlusion in more distal arterial branches. PRP, owing to its highly thrombogenic nature—containing platelet concentrations 2.5 to 8 times higher than normal serum—can result in more extensive ischemia [11].

## 3 Management of Ophthalmic Filler Embolism

When managing embolic vascular occlusions, the primary objective is to re‐establish blood flow and oxygen delivery to the compromised tissue. A range of therapeutic strategies has been proposed, including mechanical displacement of emboli, increasing local oxygen concentration, applying thrombolytic agents to dissolve fibrin clots, and administering substances specifically aimed at breaking down embolic material. While none of these methods have shown proven effectiveness in randomized clinical trials—largely due to the rarity of filler‐related embolic events—they remain relevant considerations, especially given the urgent need for prompt reperfusion, as supported by retinal ischemia models. Future studies will likely adopt rapid‐response protocols similar to those used in acute stroke and cardiovascular care.

Hyaluronic acid is currently the only cosmetic filler that has a specific antidote—hyaluronidase. This enzyme promotes the breakdown of hyaluronic acid through enzymatic degradation. Several studies have explored the efficacy of delivering hyaluronidase via subcutaneous, retrobulbar, or intra‐arterial injections in efforts to reverse ischemic damage [12]. When injected subcutaneously, hyaluronidase can diffuse through tissues and across arterial walls. A systematic review of 144 reported cases using hyaluronidase to treat vision loss caused by hyaluronic acid fillers demonstrated inconsistent outcomes in visual recovery, even when treatment was administered promptly after symptom onset [13].

## 4 Guideline for Retrobulbar Hyaluronidase

**FIGURE 1:**
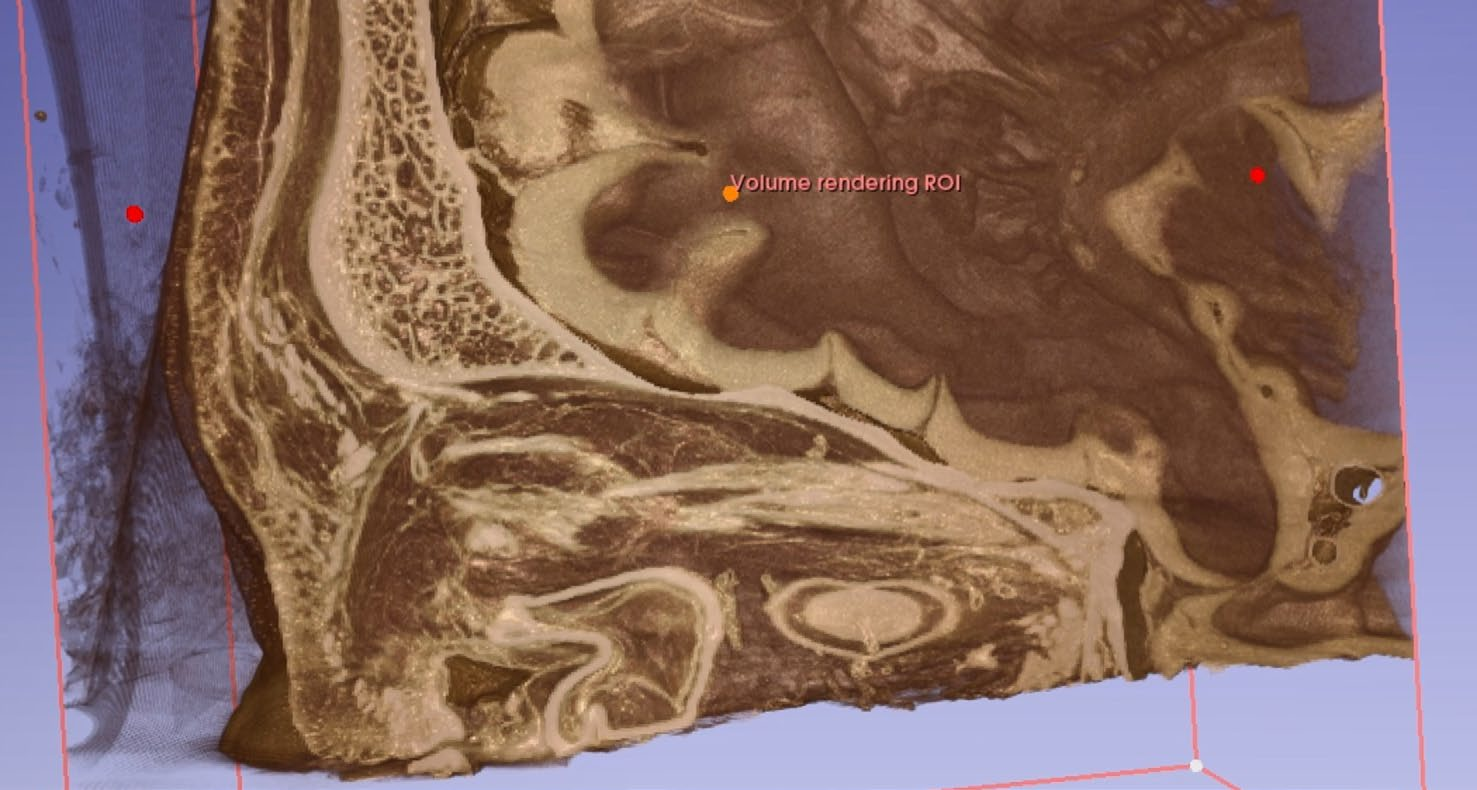
A micro‐CT image capturing the traversal of the central retinal artery into the optic canal.

When performing a retrobulbar hyaluronidase injection, the target is the region where the central retinal artery enters the optic canal (Figure 1). Dividing the orbital rim into quadrants results in superior medial, superior lateral, inferior medial, and inferior lateral divisions. Typically, approaching from the superior quadrant is not recommended, as the central retinal artery usually enters below the optic canal. Methods for approaching from the inferior medial and inferior lateral directions have been confirmed through anatomical examination.

**FIGURE 2:**
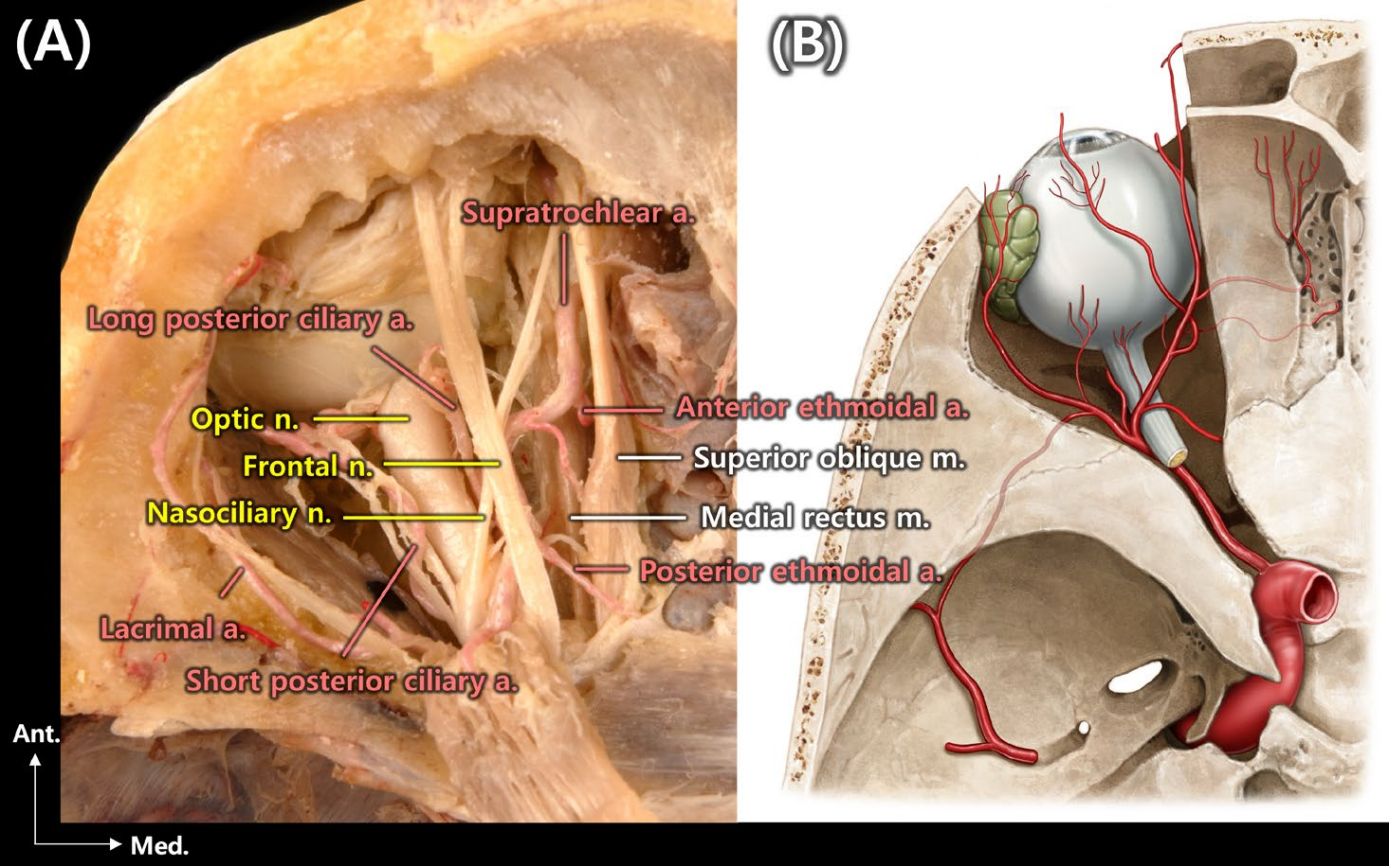
Numerous anatomical structures are depicted running medially (A), and an approach from the medial aspect carries the potential risk of causing damage to several anatomical structures (B).

Upon anatomical examination, it was observed that by scraping the orbital floor after advancing under the orbital rim below the medial canthus, a method of entry was established. The distance to the central retinal artery was found to be short. However, entering in this way involves the needle scraping the floor, and afterwards, it needs to be lifted slightly to target the optic nerve. During this process, there is currently a higher probability of puncturing the eyeball, potentially causing damage to the inferior rectus muscle. It may also be challenging to control the angle of the syringe, which could risk hitting the medial rectus (Figure 2).

When targeting the halfway point across the inferolateral quadrant of the orbit, it is possible to minimize damage to muscles, nerves, and blood vessels. The ophthalmic artery, a branch of the internal carotid artery distributed within the eye, enters the eye through the optic canal alongside the optic nerve. Positioned on the lower and lateral side of the optic nerve, it crosses above it and runs forward from the medial corner of the eye. Excluding the laterally directed short posterior ciliary artery, the inferolateral quadrant is identified as a relatively safer injection site compared to other quadrants. Notably, nerves follow a path similar to that of the arteries. Additionally, the inferolateral quadrant also has no other muscles located in the space between the lateral and inferior rectus muscles. Consequently, the halfway point across the inferolateral quadrant of the orbit is deemed the safest route for retrobulbar injections compared to other quadrants.

The standard length of the eyeball is commonly recorded as 24 mm, and the ciliary ganglion, situated laterally to the optic nerve, is consistently acknowledged to be positioned approximately 1.5 cm posterior to the globe. Accordingly, a needle length of 35 mm is deemed suitable for secure injection, facilitating access to the retrobulbar space of the eyeball. An optimal needle gauge for this purpose is identified as 22–23G. It is essential to note that a higher gauge may encounter difficulty in penetration and could potentially undergo bending.

**FIGURE 3:**
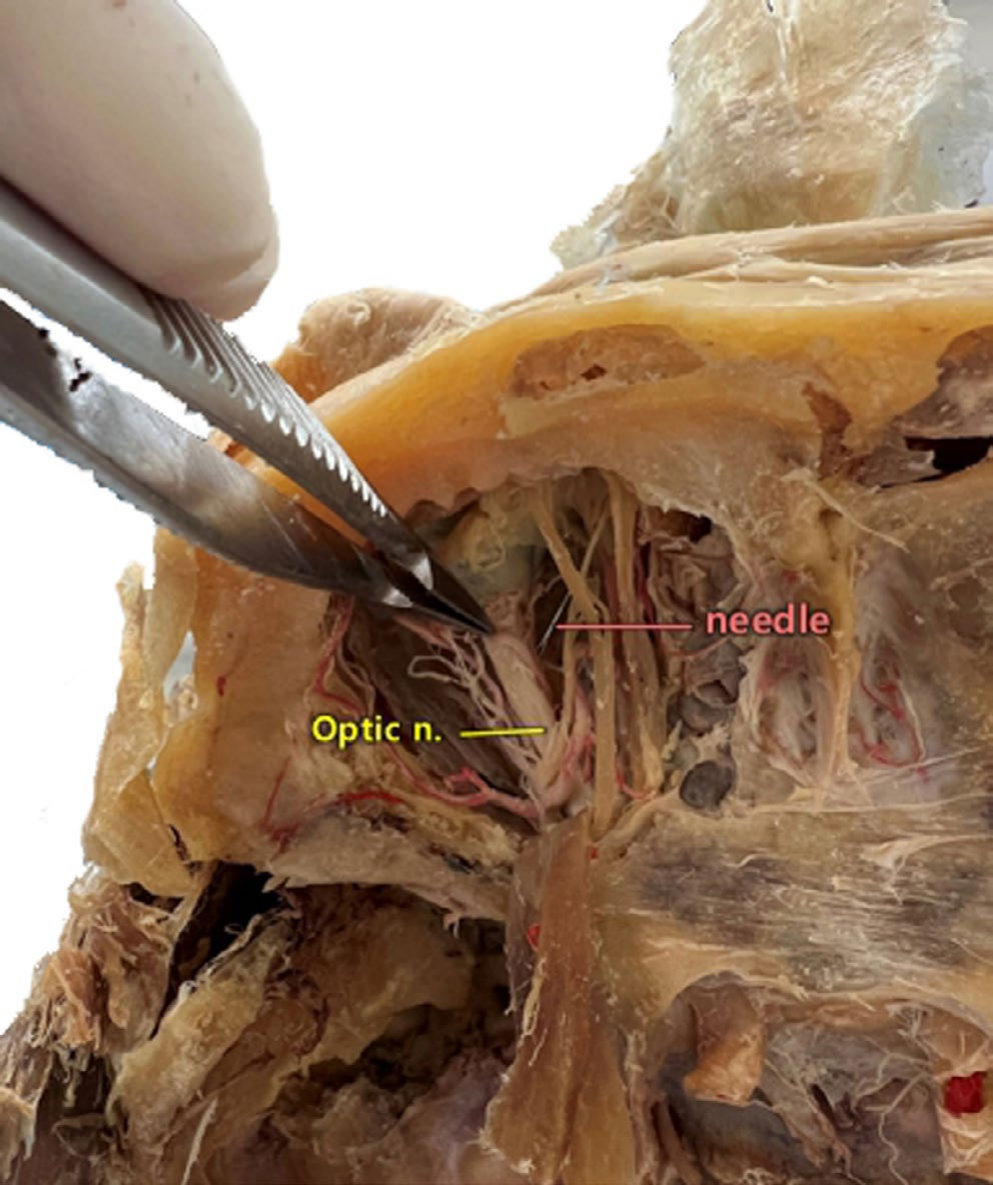
The cadaveric demonstration of a medial‐inferior approach from the orbital rim is not recommended, as it may damage anatomical structures. The recommended technique for administering hyaluronic acid injection, emphasizing the preferred direction as from the lateral inferior quadrant towards the superior medial quadrant.

In other words, establish the entry point in the inferior lateral quadrant. When initially entering, scrape the floor for about 1 cm, then lift the needle slightly and enter medially to administer the injection. More precisely, it is recommended to inject from the inferior quadrant towards the superior medial quadrant direction (Figure 3).

## 5 Discussion

Efforts have been directed towards exploring the potential of hyaluronidase in addressing ischemic damage caused by hyaluronic acid (HA) filler injection. One approach involves injecting hyaluronidase directly into the ophthalmic artery through selective angiography. While this technique has shown partial recanalization of the ophthalmic artery in some cases of HA filler‐induced vision loss, its limited accessibility due to the requirement for specialized facilities and advanced skills poses a challenge for widespread use as a rescue treatment [13, 14].

Administering retrobulbar injections of hyaluronidase in doses ranging from 1500 to 3000 IU, either once or twice, may fall short of providing a timely and effective intervention—particularly when the goal is to restore retinal artery flow within a critical four‐hour window [15, 16, 17, 18]. The lack of successful recanalization does not appear to stem from inadequate dosing, as the enzyme's half‐life within the retrobulbar space is believed to be sufficient for therapeutic action.

Nonetheless, even with adequate levels of active hyaluronidase present in the retrobulbar area, failure to re‐establish blood flow may result from limited diffusion of the enzyme into the ocular branches of the ophthalmic artery, which may be less permeable than vessels like the cadaveric facial artery. Furthermore, stagnant blood flow in the blocked branches and the presence of natural hyaluronidase inhibitors in human plasma may hinder the enzyme's ability to reach and degrade distal HA emboli after crossing into the arterial circulation.

While literature advocates retrobulbar hyaluronidase injection for treating HA filler‐induced vision loss, its effectiveness remains inconclusive, and concerns about ocular perforation risk with repeated injections prompt consideration of direct infusion into the ophthalmic artery through selective angiography [15, 18]. Acknowledging the potential need for hyaluronidase and a fibrinolytic drug to address secondary thrombosis after HA embolization, clinical studies inconsistently support this concept. Altering retrobulbar hyaluronidase dose or frequency poses unforeseeable risks to ocular tissues and the optic nerve. In summary, while hyaluronidase shows promise for HA filler‐induced vision loss, optimal delivery methods, doses, and potential combination therapies necessitate further investigation in clinical studies.

A study exploring retrobulbar hyaluronidase injection as a treatment for vision loss due to HA gel fillers found that hyaluronidase, when applied retrobulbarly, cannot cross the dural sheath of the optic nerve. Consequently, the study suggests limited efficacy of retrobulbar hyaluronidase injection in alleviating vision loss from HA gel‐induced central retinal artery occlusion [16, 19].

Navarro‐Hernandez et al.'s study focused on the evidence‐based efficacy of retrobulbar hyaluronidase injection for treating visual loss from periocular cosmetic filler injections, especially those with accidental intravascular HA injection. Improvement was observed in only 3 out of 17 cases treated with retrobulbar hyaluronidase, emphasizing the need for additional studies to establish its efficacy for blindness caused by HA [20].

The protocol presented by Carruthers et al. [8] offers a structured method for performing retrobulbar injections in cases of sudden vision loss caused by deep filler injection, particularly in situations where an ophthalmologist is not immediately accessible. According to the described technique, the procedure begins with the application of topical anesthesia—such as 0.5% tetracaine or another suitable anesthetic eye drop—for transconjunctival access. If the injection is performed through the skin, 0.1–0.2 mL of 1% lidocaine is injected into the lower eyelid, positioned midway between the center and lateral aspect of the lid.

**FIGURE 4:**
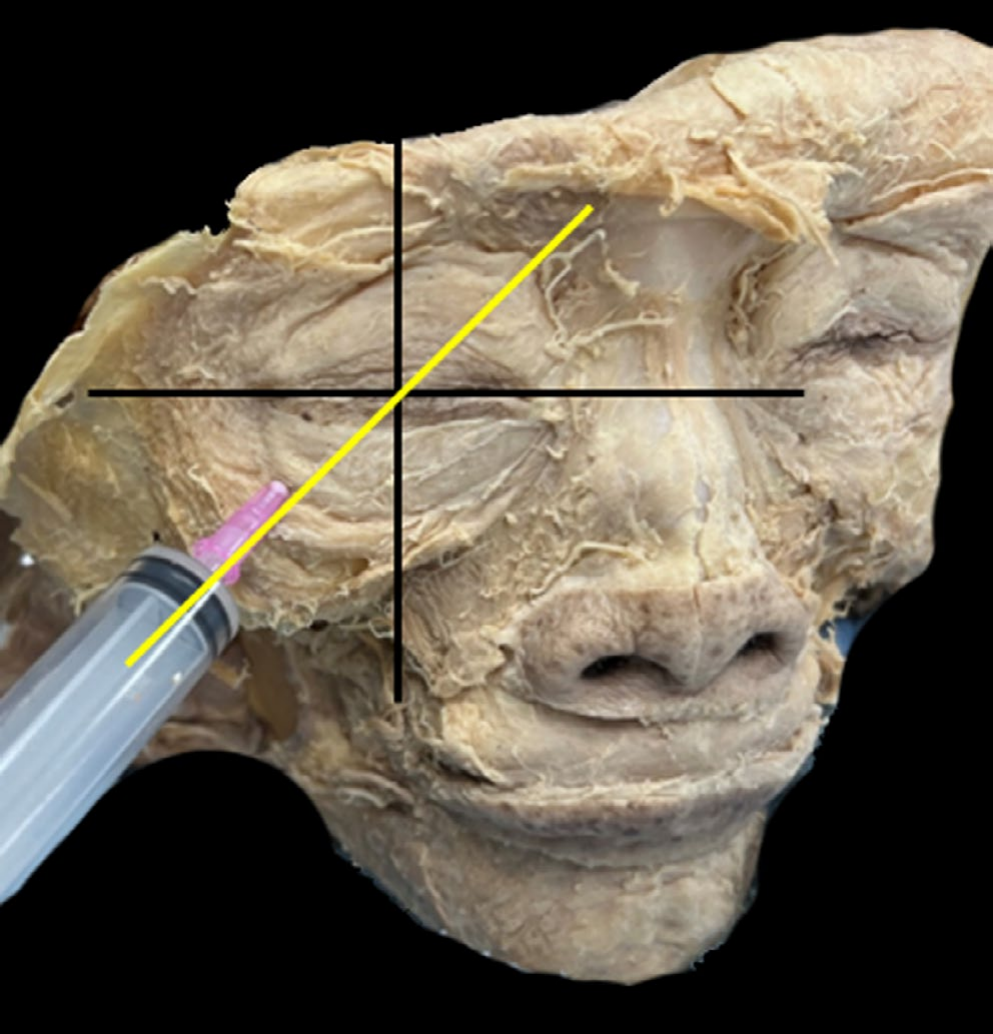
The sagittal section of the lateral part of the orbital rim (A) and observed from the transverse section (B). The use of a 35 mm needle is considered appropriate for secure injection, providing access to the retrobulbar space of the eyeball. The optimal needle gauge for this purpose is identified as 22–23G. It is crucial to emphasize that a higher gauge may encounter difficulties in penetration and is susceptible to potential bending.

**FIGURE 5:**
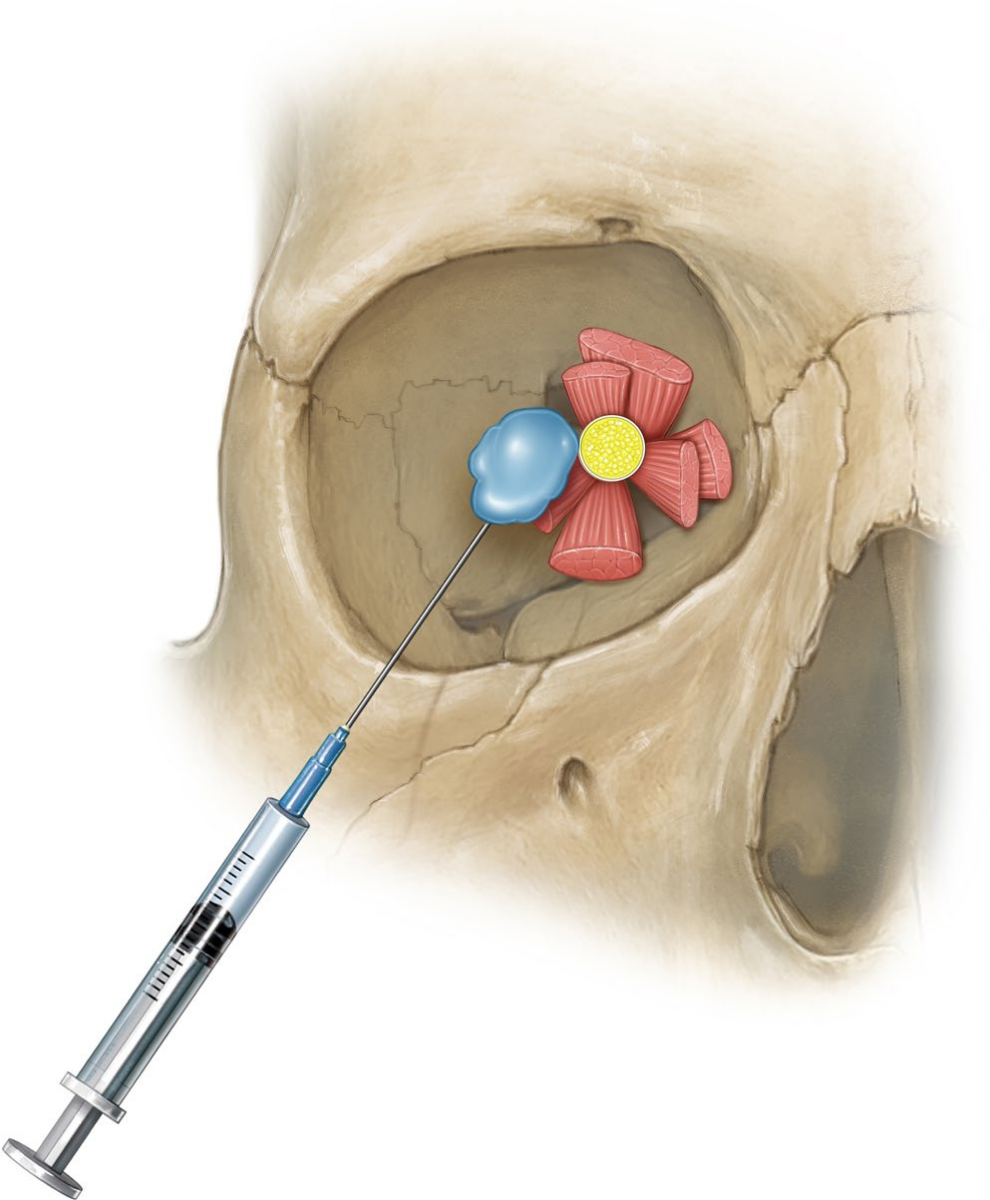
Lateral view illustrating the needle trajectory and anatomical approach for retrobulbar hyaluronidase injection.

**FIGURE 6:**
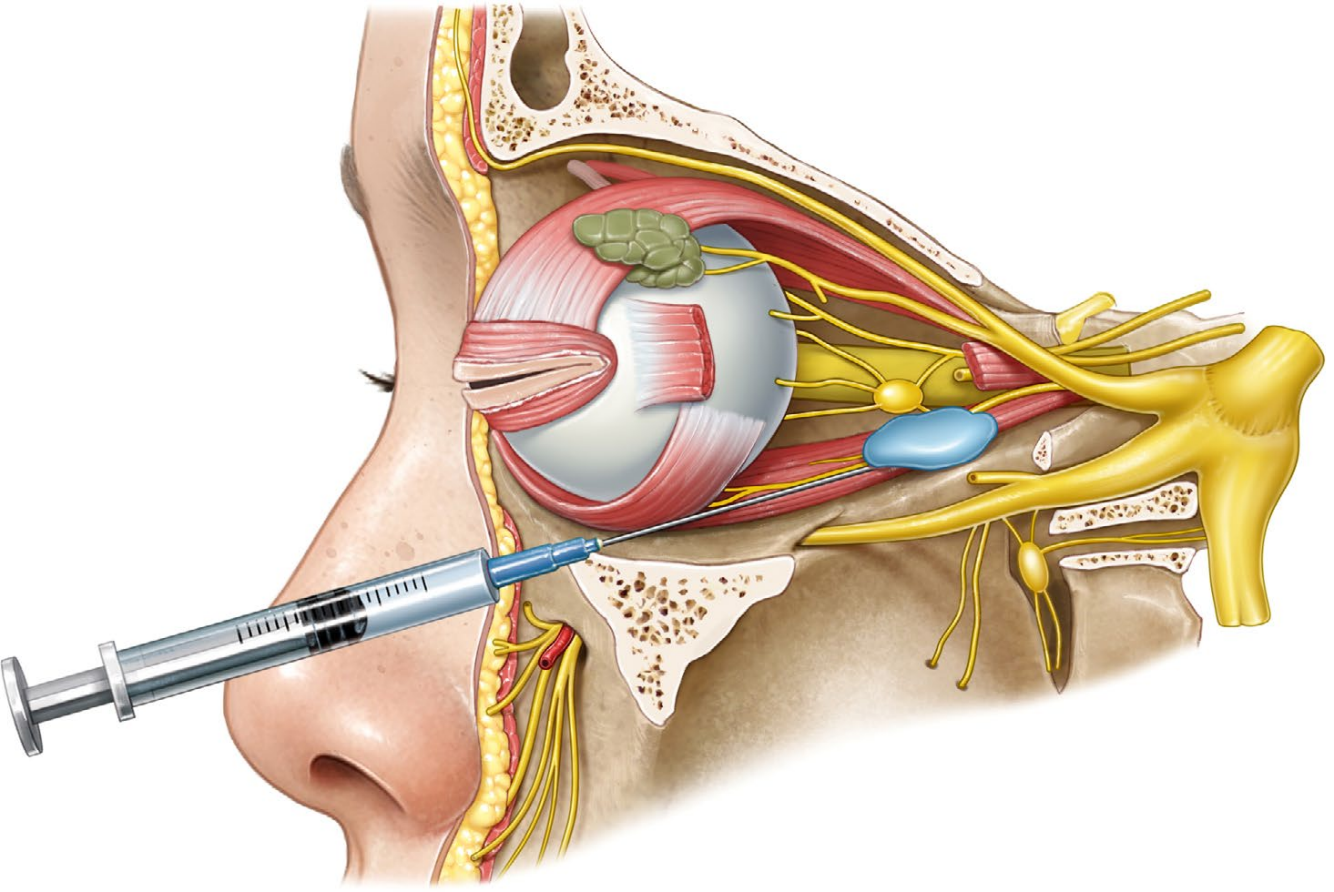
Frontal view demonstrating the entry point and direction of hyaluronidase delivery into the retrobulbar space.

Next, a 25‐gauge, 1.5‐in. needle preloaded with at least 500 units of hyaluronidase is used. The needle is carefully inserted into the inferotemporal quadrant of the orbit, initially advanced parallel to the orbital floor, and then redirected posteriorly towards the back of the globe—while taking care to avoid contact with the eye or optic nerve. The hyaluronidase is then gently injected into the retrobulbar space, with volumes ranging from 3 to 8 mL, adjusted based on the patient's orbital anatomy. However, while this method is comprehensive, it carries the risk of injury and may be technically difficult to perform in practice (Figures 4, 5, 6).

The rising popularity of injectable cosmetic fillers has been accompanied by a growing number of reports of vision loss caused by inadvertent filler embolization into the ophthalmic artery, resulting in ocular ischemia. However, despite this emerging concern, there are currently no standardized clinical guidelines recommending retrobulbar hyaluronidase injections to treat filler‐induced ischemic events.

This study seeks to systematically examine the anatomical factors relevant to hyaluronidase administration in the context of central retinal artery occlusion, aiming to re‐establish blood flow and restore visual function following ischemic complications. By filling a notable gap in current clinical recommendations, the study intends to offer meaningful guidance that may enhance the management of filler‐related vascular events. The establishment of precise anatomical protocols may improve both the safety and effectiveness of hyaluronidase use, ultimately contributing to relatively safer and more reliable outcomes in aesthetic filler treatments.

## 6 Limitations and Guideline Considerations

Despite the anatomical rationale proposed in this study, it is important to acknowledge that current international expert consensus and clinical guidelines—including those from the Aesthetic Surgery Journal (ASJ) and the European Academy of Facial Plastic Surgery (EAFPS)—generally discourage the use of retrobulbar hyaluronidase injection due to limited evidence of clinical efficacy and the high risk of severe complications, such as globe perforation or optic nerve injury. These protocols recommend that such procedures be performed only by, or under the direct supervision of, ophthalmologists or interventional radiologists. Our proposal is intended to enhance anatomical understanding rather than to advocate widespread clinical implementation. Therefore, any application of this technique must be approached with extreme caution and within a multidisciplinary context. Further clinical validation and consensus building are needed before adoption into routine practice.

## Author Contributions

All authors have reviewed and approved the article for submission. Conceptualization, Kyu‐Ho Yi. Writing – original draft preparation: Kyu‐Ho Yi, Jin‐Hyun Kim. Writing – review and editing: Kyuho‐Yi, Han Earl Lee. Visualization: Soo‐Bin Kim, Jong Keun Song. Supervision: Kyuho Yi.

## Consent

Consent was received from the families of the deceased patients before beginning the dissection.

## Conflicts of Interest

The authors declare no conflicts of interest.

## Data Availability

The data that support the findings of this study are available from the corresponding author upon reasonable request.
